# DNA Display III. Solid-Phase Organic Synthesis on Unprotected DNA

**DOI:** 10.1371/journal.pbio.0020175

**Published:** 2004-06-22

**Authors:** David R Halpin, Juanghae A Lee, S. Jarrett Wrenn, Pehr B Harbury

**Affiliations:** **1**Department of Biochemistry, Stanford University School of MedicineStanford, California, United States of America; **2**Department of Chemistry, Stanford University School of Humanities and SciencesStanford, CaliforniaUnited States of America

## Abstract

DNA-directed synthesis represents a powerful new tool for molecular discovery. Its ultimate utility, however, hinges upon the diversity of chemical reactions that can be executed in the presence of unprotected DNA. We present a solid-phase reaction format that makes possible the use of standard organic reaction conditions and common reagents to facilitate chemical transformations on unprotected DNA supports. We demonstrate the feasibility of this strategy by comprehensively adapting solid-phase 9-fluorenylmethyoxycarbonyl–based peptide synthesis to be DNA-compatible, and we describe a set of tools for the adaptation of other chemistries. Efficient peptide coupling to DNA was observed for all 33 amino acids tested, and polypeptides as long as 12 amino acids were synthesized on DNA supports. Beyond the direct implications for synthesis of peptide–DNA conjugates, the methods described offer a general strategy for organic synthesis on unprotected DNA. Their employment can facilitate the generation of chemically diverse DNA-encoded molecular populations amenable to in vitro evolution and genetic manipulation.

## Abstract 2

A method is presented that makes possible the use of standard organic reaction conditions and common reagents in the presence of unprotected DNA, an important step in enabling DNA-directed chemical synthesis and drug discovery

## Introduction

A number of strategies have been proposed recently to enable the in vitro selection and evolution of chemical libraries (Harbury and Halpin 2000; Gartner and Liu 2001). These new approaches to molecular discovery rely upon DNA-directed synthesis, whereby a physical linkage is established between DNA “genes” and respective nonbiological synthetic “gene products.” Such encoded syntheses proceed through a repeated series of two associated steps: reading of sequence information in the DNA and execution of an appropriate chemical transformation. A fundamental obstacle in all cases is covalently constructing a synthetic entity in the presence of unprotected DNA.

One approach takes advantage of hybridization to induce proximity between reactants covalently attached to oligonucleotides. “Reading” is accomplished by the hybridization of the reactant conjugates to a DNA template, whereas synthetic execution results from the reactants being positioned closely together. The strategy has been demonstrated for several types of chemistries (Orgel 1995; Bruick et al. 1996; Xu et al. 2001; Gartner et al. 2002a; Li et al. 2002). However, these proximity methods are necessarily limited to reaction conditions compatible with DNA hybridization, precluding a large number of potential chemical transformations. Moreover, only synthetic reagents that have been precoupled to DNA can be used.

Rather than tailoring reactions to the narrow window of hybridization conditions, DNA reading and chemical transformation can be carried out in chronologically distinct steps (Halpin and Harbury 2004b). The DNA is first physically partitioned into subpools by hybridization, accomplishing the reading step. An appropriate reaction is then carried out on each physically separate subpool. As such, the chemical process can take place under DNA-denaturing conditions, permitting the use of organic solvents, high pH, and elevated temperature. Although DNA exhibits limited solubility in nonaqueous solvents, its immobilization on a solid phase can be exploited to access such environments. With a solid-phase chemistry approach, a large existing body of known chemical transformations becomes accessible to DNA-encoded synthesis. Solid-phase approaches also facilitate rapid, efficient, small-scale (nanomole) syntheses. Reagents can be used in vast excess, postreaction work-up involves only washing of the solid phase, and solution-phase manipulation steps that lead to material losses are avoided. However, the conventional attachment of the small molecule to a solid phase through an irreversibly cleavable linker is not adequate, because DNA must repeatedly come on and off the solid phase during reading steps. Here we report a detailed strategy for carrying out solid-phase organic chemistry on unprotected DNA that is suitable for encoded library synthesis by a partitioning approach. Furthermore, we demonstrate a number of tools for adapting new chemistries, and we use those tools to develop comprehensive methods for peptide synthesis on unprotected DNA.

## Results

### Solid-Phase Synthesis Resin

We first had to choose a solid-phase material that exhibited several critical properties: reversible, efficient binding and release of unprotected DNA; robust solvent integrity; and resistance to chemical modification. The first requirement narrowed our focus to resins that noncovalently bind DNA. A number of resins were tested and excluded due to poor bind–release properties (diethylaminoethyl [DEAE] silica, Macro-Prep ceramic hydroxyapatite, and quaternary amine anion exchange resins). Others exhibited extensive compression in organic solvent (Sephacryl S-1000, macroporous methacrylate) or poor reswelling during organic to aqueous solvent transitions (Poros 50 HQ). Reverse-phase resins were excluded because they would presumably not retain DNA in many organic solvents.

**Figure 1:**
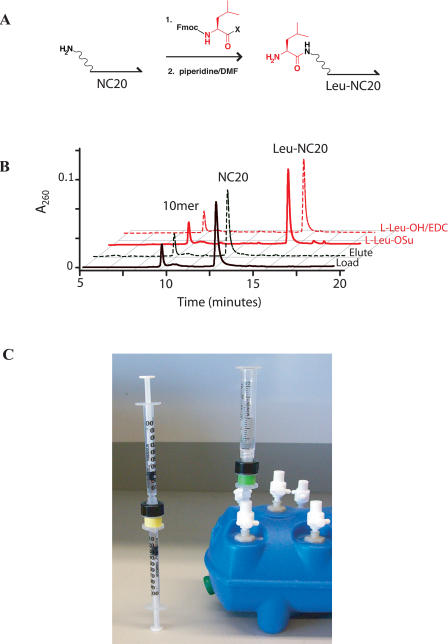
Peptide Coupling to DNA Supports (A) Fmoc-based peptide coupling reaction to an aminated 20-base oligonucleotide (NC20) where X represents a succinimidyl or EDC/HOAt-activated ester. (B) HPLC chromatograms of a nonaminated 10-base (10mer) and an aminated 20-base (NC20) oligonucleotide. HPLC traces show DEAE column load (solid black) and elute (broken black). DEAE column elutes after succinimidyl ester–mediated (solid red) or EDC/HOAt-mediated (broken red) Fmoc-Leu coupling and Fmoc deprotection are shown. The resulting amino acid–DNA conjugate is denoted (Leu-NC20). (C) Chemical transformations are carried out using small DEAE Sepharose columns and syringes (left). Washes are facilitated by a vacuum manifold with chemically resistant stopcocks (right).

DEAE Sepharose, a tertiary amine anion exchange resin, exhibited excellent bind and release properties. By a high performance liquid chromatography (HPLC) assay, oligonucleotides were immobilized and eluted quantitatively in small volumes (Figure 1). Single-stranded DNA (ssDNA) molecules as long as 340 bases were also bound and eluted with high efficiency (data not shown). No severe resin compression was observed using a number of solvents, including H_2_O, methanol (MeOH), dimethyl sulfoxide, *N*,*N*-dimethylformamide (DMF), ethyl acetate, and dichloromethane. Lastly, Sepharose has been used previously as a material for solid-phase synthesis (Tegge and Frank 1997; Nakaie et al. 2003). All subsequent work was carried out with DEAE Sepharose.

### Peptide Chemistry

**Figure 2:**
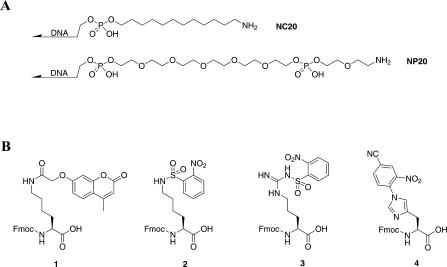
DNA Support Structure and Modified Amino Acids (A) Peptide synthesis is carried out on DNA modified with a 5′ C12 amino (NC20) or a 5′ PEG amino (NP20) linker. (B) Fluorescent lysine derivative (compound 1, Fmoc-Lys[coumarin]-OH) and BME/DBU labile protecting groups for lysine (compound 2, Fmoc-Lys[Ns]-OH), arginine (compound 3, Fmoc-Arg[Ns]-OH), and histidine (compound 4, Fmoc-His[CNP]-OH).

We studied 9-fluorenylmethyoxycarbonyl (Fmoc)–based peptide synthesis because it has a well-established solid-phase precedent and offers a challenge in diverse chemical functionality (Figure 1A). As a DNA support, we chose a 20-base oligonucleotide modified with a 5′ primary amine (NC20; Figure 2) so that coupling reactions could be readily assayed by HPLC. NC20 and an unmodified 10-base oligonucleotide control (10mer) were bound to a DEAE Sepharose column, washed with DMF, and incubated with an Fmoc–amino acid succinimidyl ester solution in a closed system (see Figure 1C). A solution of 20% piperidine in DMF was then used to remove the Fmoc group. The DNA was eluted and compared to starting material via an HPLC mobility assay (Figure 1B). Coupling and Fmoc-deprotection proceeded with high efficiency (95% or greater) in 30 min. Furthermore, the internal control oligonucleotide was unmodified and fully recovered, ruling out nonspecific DNA modification. Further experiments indicated that addition of the first amino acid proceeds more efficiently than subsequent additions. We therefore optimized coupling for the addition of a second amino acid to an oligonucleotide already acylated with leucine (Leu-NC20). Nearly quantitative coupling was observed for all amino acids, and all conjugates were verified by matrix-assisted laser desorption/ionization mass spectrometry (MALDI-MS) (Table 1). However, the succinimidyl esters did not efficiently acylate proline residues.

**Table 1:**
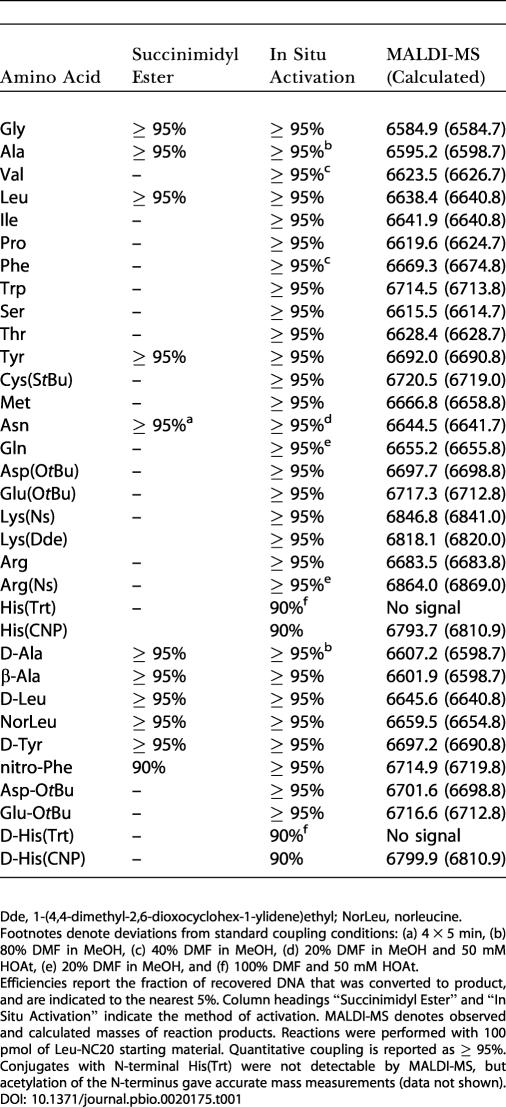
Amino Acid Coupling Efficiencies to Leu-NC20

Dde, 1-(4,4-dimethyl-2,6-dioxocyclohex-1-ylidene)ethyl; NorLeu, norleucine

Footnotes denote deviations from standard coupling conditions: (a) 4 × 5 min, (b) 80% DMF in MeOH, (c) 40% DMF in MeOH, (d) 20% DMF in MeOH and 50 mM HOAt, (e) 20% DMF in MeOH, and (f) 100% DMF and 50 mM HOAt

Efficiencies report the fraction of recovered DNA that was converted to product, and are indicated to the nearest 5%. Column headings “Succinimidyl Ester” and “In Situ Activation” indicate the method of activation. MALDI-MS denotes observed and calculated masses of reaction products. Reactions were performed with 100 pmol of Leu-NC20 starting material. Quantitative coupling is reported as ≥ 95%. Conjugates with N-terminal His(Trt) were not detectable by MALDI-MS, but acetylation of the N-terminus gave accurate mass measurements (data not shown)

We next explored the possibility of in situ activation for peptide coupling. In a first pass, 1-(3-[dimethylamino]propyl)-3-ethylcarbodiimide hydrochloride (EDC) out-performed other activating reagents examined; other reagents gave poor coupling yields (benzotriazole-1-yl-oxy-tris-pyrrolidino-phosphonium hexafluorophosphate [PyBOP]), resulted in the formation of undesired side products (2-[1H-benzotriazole-1-yl]-1,1,3,3-tetramethyluronium tetrafluoroborate [TBTU]), or led to poor recovery of DNA (dicyclohexylcarbodiimide [DCC] and diisopropylcarbodiimide [DIC]). Thirty-minute EDC coupling reactions were typically less than 50% efficient without the addition of acylation catalysts such as *N*-hydroxysuccinimide, 1-hydroxy-7-azabenzotriazole (HOAt), or *N*-hydroxybenzotriazole (Nozaki 1997). Of these, HOAt was superior, bringing coupling efficiencies above 90%. A number of reaction solvents were examined, including H_2_O, DMF, MeOH, isopropanol, dichloromethane, and mixtures thereof. In general, MeOH gave the best results, with DMF only slightly worse, followed by isopropanol then H_2_O. For most amino acids, exceptional coupling was achieved with 30-min coupling times using 50 mM Fmoc–amino acid-OH, 50 mM EDC, and 5 mM HOAt (see Figure 1B; Table 1, Figure S1). Importantly, amino acids activated in situ with EDC acylated proline efficiently. We observed equally efficient coupling using a more hydrophilic polyethylene glycol (PEG) linker (NP20; see Figure 2), which might be better suited for biological applications and in vitro selections (Halpin and Harbury 2004b).

To examine whether the coupling conditions generalized to longer DNA fragments, we used an aminated 340-base ssDNA as the support. After coupling, the eluted amino acid–DNA conjugates were digested with nuclease P1, a 3′-to-5′ exonuclease that cleaves all but the 5′ phosphodiester bond of our ssDNA constructs. The 5′-terminal nucleotide, which maintains the linker and synthetic peptide product, was separated from the other nucleoside monophosphates by HPLC and verified by electrospray ionization mass spectrometry. Amino acid coupling to the 340-base ssDNA proceeded with efficiencies comparable to those observed with oligonucleotides (data not shown). In some cases, the sensitivity of this HPLC assay was increased using fluorescence detection. For these experiments, we synthesized a fluorescent lysine derivative, Fmoc-Lys(coumarin)-OH (see Figure 2B, compound 1), that was incorporated as the C-terminal amino acid. The fluorescence signal allowed sensitive monitoring of subnanomolar quantities of product.

**Figure 3:**
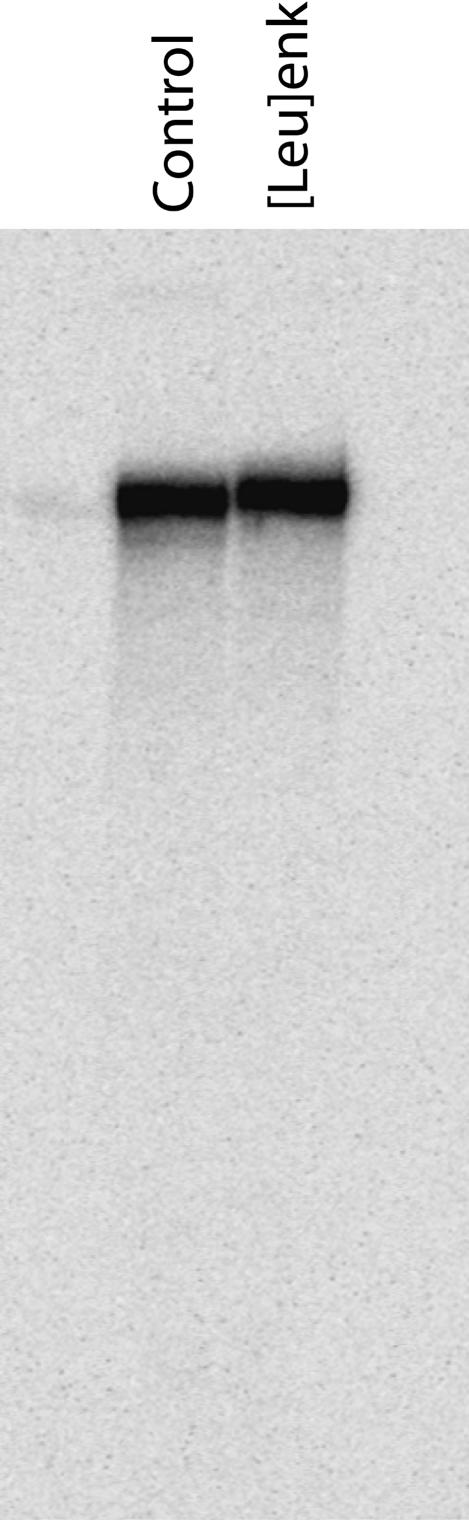
Peptide–DNA Conjugate As Template for DNA Synthesis 5′ PEG amino-modified 340-base ssDNA was loaded onto two DEAE Sepharose columns. The pentapeptide [Leu]enkephalin was synthesized on one column using EDC/HOAt and Fmoc–amino acids. The DNA was eluted, desalted, and used as template for radiolabeled primer extension reactions. Denaturing polyacrylamide gel electrophoresis analysis of reaction products shows no difference between ssDNA (control) and [Leu]enkephalin–ssDNA ([Leu]enk) templates.

By mass spectrometry and HPLC criteria, the peptide synthesis procedures did not damage oligonucleotides. As a more stringent test for possible DNA damage, we synthesized a pentapeptide on a 340-base ssDNA support and examined the ability of the conjugate to act as a template for primer extension. Polyacrylamide gel electrophoresis analysis of the radiolabeled extension products showed no truncated fragments (Figure 3), providing further evidence that the synthetic procedures are DNA-compatible.

### Side Chain Protection

Typically, acid-labile groups are used to protect reactive amino acid side chains in Fmoc peptide synthesis. Given the instability of DNA in strong acids, it was necessary to identify alternate protecting group strategies. In all cases, the protecting groups were required to be stable during peptide coupling procedures and removed under alternate DNA-compatible conditions.

The carboxylic acid side chains of aspartic and glutamic acid are usually protected as esters during Fmoc peptide synthesis. Sterically bulky esters are required to suppress piperidine-induced imide formation, which leads to undesired side chain peptide bonds. We discovered that the conventional *tert*-butyl (*t*Bu) esters, which are normally cleaved with trifluoroacetic acid, could be removed at pH 6.5 in an aqueous solution at 70 °C. This gentle condition offers a convenient approach for acid deprotection.

To verify that the thermolytic *tert*-butyl ester deprotection did not proceed through intramolecular imide formation, we coupled either Fmoc-Asp(*t*Bu)-OH or Fmoc-Asp-O*t*Bu to Leu-NC20, followed by Fmoc-Phe-OH. The main and side chain isomers of these tripeptide–DNA conjugates were resolved by HPLC after removal of the *t*-butyl group. Interconversion of the isomers during deprotection was undetectable (less than 5%). When the experiment was repeated using a 10 mM NaOH solution for *tert*-butyl ester deprotection (where imide formation would be expected), interconversion of the side and main chain isomers was observed. These results indicate that the thermolytic deprotection maintains the regiochemistry of the initial peptide bonds. At this time, we have little other data that speak to the mechanism of deprotection.

Protection of the primary amine side chain of lysine prevents the formation of branched peptides. 2-Nitrobenzenesulfonamide (nosylamide) protection was particularly attractive as a means for lysine protection because the protecting group is base-stable and removed under conditions known to be DNA-compatible (Fukuyama et al. 1995). The lysine side chain was nosylated in good yield to produce Fmoc-Lys(Ns)-OH (see Figure 2B, compound 2). The nosyl (Ns) group was removed quantitatively from Lys(Ns)-containing peptide–DNA conjugates by β-mercaptoethanol (BME)/1,8-diazabicyclo[5.4.0]undec-7-ene (DBU) in a DMF solution at 60 °C.

Arginine does not absolutely require side chain protection (Table 1). However, we observed a marked decrease in coupling efficiency as multiple unprotected arginine residues were added at adjacent positions in a synthetic peptide (first Arg approximately 95%; second Arg approximately 50%; third Arg less than 10%). These difficult transformations were accomplished in high yield by repeated high-temperature couplings (4 × 30 min, 37 °C). However, protection of the guanidino group of arginine would offer a more general solution. Given that the conventional arginine protecting groups are sulfonamides, we speculated that nosylamide protection could be applied. We synthesized Fmoc-Arg(Ns)-OH (see Figure 2B, compound 3) from Boc-Arg-OH and found that it coupled well (Table 1) and was quantitatively deprotected under the BME/DBU conditions.

Side chain protection of histidine is essential to prevent acyl-transfer reactions and to suppress L,D-racemization. We found that the trityl (Trt) group of His(Trt) is rapidly removed under the thermolytic conditions used to deprotect *tert*-butyl esters. However, the trityl group is not ideal in aqueous conditions because of its hydrophobicity and extreme lability at high temperatures. To offer a more robust solution, we sought an H_2_O-compatible histidine protecting group. The 2,4-dinitrophenyl group, widely used in Boc peptide synthesis, is more hydrophilic than trityl and is removed under the nosyl-deprotection conditions. Unfortunately, 2,4-dinitrophenyl is not stable to the piperidine used for Fmoc removal (Garay et al. 1997). After testing a number of nitro-phenyl derivatives, we found that the 4-cyano-2-nitro-phenyl (CNP) group exhibits the appropriate reactivity. We synthesized Fmoc-His(CNP)-OH (see Figure 2B, compound 4) in one step from Fmoc-His-OH. The CNP group is stable to 20% piperidine in DMF and is deprotected fully with BME/DBU.

Histidine racemization is a well-recognized concern in peptide synthesis. To assay the extent of racemization occurring during histidine coupling, we synthesized the oligonucleotide–dipeptide conjugate His(CNP)-Ala-NC20 using either L- or D-Fmoc-His(CNP)-OH. The diasteriomeric dipeptide–oligonucleotide products were resolvable by reverse-phase HPLC. Neither L-His nor D-His coupling resulted in detectable racemization. The experiment was repeated using L- and D-Fmoc-His(Trt)-OH with the same result.

Fmoc-Cys(S*t*Bu)-OH was employed as the protected form of cysteine. The *tert*-butyl thioether coupled efficiently (Table 1) and was deprotected under the same conditions used to deprotect lysine, arginine, and histidine (CNP). Although we were unable to recover free thiol-containing peptides from our HPLC system for unknown reasons, we could alkylate the deprotected thiol side chain and recover the thioether-containing peptide–oligonucleotide conjugates.

### Polypeptide Synthesis

To characterize multistep syntheses (Gartner et al. 2002b), we prepared a number of peptide–DNA conjugates, ranging from two to 12 amino acids in length and varying in sequence and degree of side chain protection required (Protocol S1). In all cases, absolute yields of conjugates with *n* amino acids exceeded (0.9)*^n^* (Figure S2). The HPLC-purified conjugates were analyzed by Edman degradation. Peptide sequencing data were unambiguous and in full agreement with the intended synthetic peptide sequences (Table 2), ruling out side chain modifications that could not be detected by MALDI-MS.

**Table 2:**
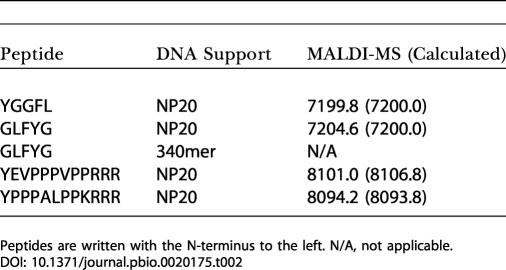
Sequenced Peptide–DNA Conjugates

Peptides are written with the N-terminus to the left. N/A, not applicable

Beyond the physical and chemical characterization of the peptide–oligonucleotide conjugates, it was important to examine their behavior in a biochemical setting. Thus, the [Leu]enkephalin pentapeptide was synthesized on an aminated 340-base ssDNA support, which was subsequently converted to duplex form. The conjugate exhibited a peptide-dependent electrophoretic mobility gel shift when incubated with the 3-E7 antibody (Halpin and Harbury 2004b), demonstrating its biochemical activity.

## Discussion

DNA-directed synthesis requires “chemical translation” (Gartner and Liu 2001; Halpin and Harbury 2004b). Rather than simply acting as a tag to report a synthetic history (Brenner and Lerner 1992), the DNA must actively determine the series of reactions that construct the molecule. No matter how this is achieved, it involves associated steps of DNA “reading” and synthetic execution. The synthetic steps must not damage the DNA and consequently compromise the reading process. Once each product is covalently attached to an amplifiable support that carries the information necessary for its synthesis, the product molecules are amenable to evolution and genetic manipulation.

The proximity approach to chemical translation uses hybridization to induce proximity-driven chemical transformations. Because the DNA “reading” and chemical execution steps are simultaneous, reactions are necessarily performed in aqueous solutions with solute, pH, and temperature conditions that promote DNA–oligonucleotide hybridization. These conditions limit the generality, efficiency, and speed of possible organic transformations.

The partitioning strategy separates the DNA reading and the chemical step, and also introduces a solid-phase format (Halpin and Harbury 2004b). The separation overcomes incompatibility between hybridization conditions and optimal reaction conditions. Synthetic transformations are carried out using standard solvents and elevated temperature. This advantage cannot be fully appreciated with solution-phase reaction formats, given the insolubility of DNA in organic solvents. Solid-phase formats, however, can take full advantage of flexibility in reaction conditions. Once DNA is bound to the solid phase, the chemical transformation, rather than DNA solubility, dictates solvent choice. We carry out transformations in H_2_O, MeOH, ethanol, isopropanol, DMF, dimethyl sulfoxide, dichloromethane, and ethyl acetate (data not shown). We also carry out reactions at high temperatures. For example, difficult peptide couplings are facilitated with elevated temperature, and the BME-mediated deprotection of lysine, arginine, histidine, and cysteine is carried out at 60 °C. We have recently used microwave-assisted methods (Lew et al. 2002) to accelerate by 100-fold alkylation reactions on DNA-linked substrates (data not shown). Standard, commercially available reagents are used to perform chemistry, and we employ them in large excess (1,000- to 1,000,000-fold) over the DNA support, facilitating rapid reactions with high yields. The peptide coupling detailed here proceeds quantitatively in less than an hour, on par with the fastest standard solid-phase peptide synthesis coupling times. With the possible exception of *tert*-butyl esters, which are slowly removed during the 72 °C step of hybridization-mediated library splitting, the peptide chemistry and protecting groups presented here are suitable for use with DNA display (Halpin and Harbury 2004b).

Another important aspect of the solid-phase method is reversible immobilization. This is an essential component of the DNA display chemical-translation cycle, where the DNA moves on and off a series of hybridization and chemistry columns (Halpin and Harbury 2004a, 2004b). In a simplistic view, we have essentially taken advantage of the polyanionic “handle” covalently attached to our synthetic substrates. The handle acts as a phase label (Curran 1998) for solid-phase extractions with anion exchange resins. Previously, soluble polymer-supported synthesis has been accomplished with PEG and fluorinated hydrocarbon purification handles (Han et al. 1995; Curran 1998). The polyanionic handle here uniquely accommodates both liquid- and solid-phase chemical steps.

Our approach offers a general tool for derivatization of DNA. Unprotected peptide–DNA conjugates have been recognized as biochemically useful reagents for over 15 years (Corey and Schultz 1987; Zuckermann and Schultz 1988; Allinquant et al. 1995; Tong et al. 1995; Troy et al. 1996). The methods described here are efficient, rapid, and inexpensive, and they utilize DNA of synthetic or enzymatic origin, offering advantages over previously reported techniques (Robles et al. 1999; Stetsenko and Gait 2000; Debethune et al. 2002).

Importantly, the protocols are not inherently limited to peptides. We have designed a set of tools for the adaptation of new chemistries. Reaction conditions are rapidly examined and optimized with oligonucleotides using HPLC mobility assays and MALDI-MS analysis. Nuclease P1 digestion facilitates the characterization of reactions on long DNA fragments and improves chromatographic and mass spectral resolution of synthetic products. MALDI-MS, primer extension analysis, and DNA sequencing reveal the presence of chemistry-induced DNA damage. We have used these tools to develop highly efficient protocols for solid-phase *N*-substituted polyglycine (“peptoid”) submonomer synthesis on unprotected DNA (data not shown). The chemistry used for peptoid synthesis is entirely different from peptide chemistry, illustrating the generality of the strategy. The potential for adapting other chemistries is essentially limitless. Wittig reactions, azide reductions, 1,3 dipolar cycloadditions, reductive aminations, Heck couplings, and a wide variety of other useful chemical transformations have been carried out in the presence of unprotected DNA without modification of DNA (Bruick et al. 1996; Xu et al. 2001; Gartner et al. 2002a; Li et al. 2002). Each could be used to synthesize and evolve interesting and potentially useful small molecule–DNA conjugate libraries.

Our results demonstrate a robust method for solid-phase organic synthesis on unprotected DNA supports. Taken with the chemical-translation and DNA-manipulation strategies detailed elsewhere (Halpin and Harbury 2004a, 2004b), they facilitate a physical linkage between “genes” and synthetic “gene products” that is generalizable with respect to chemistry. The establishment of such a genetic underpinning to synthetic chemistry makes possible in vitro selection-based molecular discovery strategies for wholly abiotic molecular populations.

## Materials and Methods

### 

#### Materials.

Fmoc amino acids were purchased from Novabiochem (La Jolla, California, United States), Chem-Impex International (Wood Dale, Illinois, United States), or Fluka (Basel, Switzerland). EDC was purchased from Omega Chemical (Levis, Quebec, Canada). *N*-hydroxysuccinimide was purchased from Chem-Impex. HOAt was purchased from Millipore (Billerica, Massachusetts, United States). Nuclease P1 (#27–0852-01), DEAE Sepharose Fast Flow (#17–0709-01), and Medium Grade G-25 Sephadex (#17–0033-01) were purchased from Pharmacia-LKB Technology (Uppsala, Sweden). Xba1 was purchased from New England Biolabs (Beverly, Massachusetts, United States). DEAE Sepharose columns were poured in Empty TWIST synthesis columns (#20–0030, Glen Research, Sterling, Virginia, United States). Kendall Monoject syringes (#1180100555, Kendall, Walpole, Massachusetts, United States) and a Promega (Madison, Wisconsin, United States) manifold with chemically resistant PFTE stopcocks (#121–0009, Argonaut, Foster City, California, United States) were used. All other chemical reagents or solvents were purchased from either Sigma-Aldrich (St. Louis, Missouri, United States) or Fisher Scientific International (Hampton, New Hampshire, United States).

The internal control 10-base oligonucleotide had the sequence CGGACTAGAG. The reactive 20-base oligonucleotides had the sequence H_2_N-X-AGCAGGCGAATTCGTAAGCC, where X represents a C12 linker (NC20) or a longer PEG linker (NP20). NC20 was synthesized using the Glen Research 5′-Amino-Modifier C12 (#10–1922). NP20 was synthesized using the Glen Research Spacer Phosphoramidite 18 (#10–1918) followed by the 5′-Amino-Modifier 5 (# 10–1905).

#### Reverse-phase HPLC assay.

Coupling reactions were monitored by HPLC mobility shift using a C18 analytical column (Microsorb, Varian, Palo Alto, California, United States) and UV detection at 260 nm and 280 nm (Spectra FOCUS, Thermo Separation Products, San Jose, California, United States). Linear gradients from 0%–90% acetonitrile in 100 mM triethylammonium acetate (pH 5.5) were employed. Coupling efficiencies (recovered product DNA/ total recovered DNA) and yields (recovered product DNA/total starting material DNA) were determined by integration of elution peaks from the 260-nm channel. Chromophores added or removed during reactions cause changes in extinction coefficients less than the sensitivity (5%) of our HPLC assay (for NC20/NP20 ɛ_260nm_ ≈ 224.5 mM^−1^cm^−1^) and were not considered in efficiency and yield determination. Reaction products were collected, concentrated to approximately 50 μM using centrifugal evaporation, and desalted over G-25 Sephadex. A mixture of 1 μl of desalted oligonucleotide and 1 μl of a freshly prepared saturated matrix solution was spotted on a matrix-assisted laser desorption/ionization target and allowed to air dry before mass spectrometry analysis. The matrix solution was made from 250 μl of H_2_O, 250 μl of acetonitrile, 25 mg of THAP, and 10 mg of ammonium tartrate. Peptide sequences (five or more amino acids) were verified by Edman degradation peptide sequencing.

#### Nuclease P1 assay.

DEAE elute buffer containing the peptide–DNA conjugates was neutralized and brought to 100 mM sodium acetate (pH 5.2) and 400 μM ZnSO_4_. Then 1 μg of nuclease P1 was added, and the mixture was incubated at 37 °C for 30 min. The entire reaction mixture was directly injected onto a C18 reverse-phase HPLC column and analyzed using linear gradients from 0%–90% acetonitrile in 10 mM ammonium acetate (pH 5.2). Yields were determined by integration of elution peaks from the 260-nm channel, using the P1 digestion product of unreacted starting material as a reference. Approximately 1 nmol of material was required for accurate UV detection. Products were collected, concentrated by centrifugal evaporation, and applied to a C18 SepPak cartridge (#WAT023590, Waters, Milford, Massachusetts, United States) in 25 mM triethylammonium acetate (pH 5.5). The cartridge was washed with 3 ml of 25 mM triethylammonium acetate (pH 5.5) and 1 ml of H_2_O. The products were eluted with 1 ml of 50/50 MeCN/H_2_O, concentrated to 100 μl by centrifugal evaporation, and analyzed by electrospray ionization mass spectrometry. For coumarin-labeled products, fluorescence was monitored (320 nm excitation/380 nm emission) with a scanning fluorescence detector (Thermo Separation Products, FL2000), and less than 50 pmol of material was necessary for accurate fluorescence detection.

#### Primer extension assay.

A 5′-aminated 340-base ssDNA support was generated as described (Halpin and Harbury 2004a). After loading the support onto DEAE Sepharose, the pentapeptide [Leu]enkephalin (Tyr-Gly-Gly-Phe-Leu) was synthesized using EDC chemistry. The resulting peptide–DNA conjugate was desalted over reverse-phase cartridge (Halpin and Harbury 2004a) and used as a template for primer extension (Halpin and Harbury 2004b). The radiolabeled duplex product was digested with Xba1, subjected to denaturing polyacrylamide gel electrophoresis, exposed to a phosphorimager cassette, and imaged on a Typhoon 8600 (dynamic range 10^5^/pixel). The intensity of full-length control and [Leu]enkephalin bands were similar to within 1% (S/N approximately 600). Upon peak integration along the entire lane, the full-length band represented a similar percentage of total intensity in the control (83%) and [Leu]enkephalin (81%) samples. The data suggest that, in the worst case, 3% of the DNA could have been modified during the course of peptide synthesis.

#### Resin loading and eluting.

Approximately 250 μl of DEAE Sepharose suspension was pipetted into an empty Glen Research column housing and washed with 20 ml of H_2_O followed by 12 ml of DEAE bind buffer (10 mM acetic acid and 0.005% Triton X-100) using a syringe or a syringe barrel, a male-male luer adapter, and a vacuum manifold (see Figure 1). The DNA was loaded onto the washed chemistry column in 1 ml of DEAE bind buffer at approximately 1 ml/min. The column was then washed with 3 ml of DEAE bind buffer, followed by 500 μl of H_2_O and 3–5 ml of the solvent required for the subsequent reaction. At least 50 nmol of oligonucleotide can be loaded onto one 250-μl DEAE Sepharose column.

After the desired chemical transformations were carried out, the column was washed with 3–5 ml of the reaction solvent followed by 3–5 ml of DEAE bind buffer. The DNA was then eluted with 2 ml of DEAE elute buffer (1.5 M NaCl, 50 mM Tris-HCl [pH 8.0], and 0.005% Triton X-100) using a syringe. Long DNA molecules (340mers) were eluted with 4 ml of 1.5 M NaCl, 10 mM NaOH, and 0.005% Triton X-100 heated to 80 °C.

#### Peptide coupling: succinimidyl esters.

The following process was carried out twice. The column, with DNA bound, was washed with 3 ml of DMF. Using two syringes (see Figure 1), the column was incubated for 5 min at room temperature with a freshly prepared solution containing 225 μl of DMF, approximately 19 mg of Fmoc–amino acid-OSu, 67.5 μl of H_2_O, and 7.5 μl of diisopropylethylamine. Fmoc-Asn-OSu required four couplings rather than two to achieve quantitative yields.

After the second amino acid incubation, the column was washed with 3 ml of DMF. Fmoc deprotection was carried out as follows: 3 ml of 20% piperidine in DMF was applied to a 3-ml syringe barrel attached to the top of the column. 1.5 ml was pushed through the column, followed by a 3-min incubation. An additional 1 ml was pushed through the column, followed by a 17-min incubation. The procedure was completed with a final 3-ml DMF wash.

#### Peptide coupling: in situ activation.

The column was washed with 500 μl of H_2_O and 3 ml of MeOH and then incubated for 30 min at room temperature with a freshly prepared 500-μl solution of 50 mM Fmoc–amino acid-OH, 50 mM EDC, and 5 mM HOAt in MeOH. These conditions were derived directly from Nozaki (1997). The column was then washed with 3 ml of MeOH. Fmoc-Asn-OH and Fmoc-His(Trt)-OH required 50 mM HOAt for efficient coupling. The following amino acids couple optimally in DMF/MeOH mixtures: Fmoc-Arg(Ns)-OH, Fmoc-Asn-OH ,and Fmoc-Gln-OH (20% DMF); Fmoc-Phe-OH and Fmoc-Val-OH (40% DMF); Fmoc-Ala-OH (80% DMF); and Fmoc-His(Trt)-OH (100% DMF). These mixtures were determined primarily by solubility. Typical reactions were carried out with 100 pmol of aminated oligonucleotide (see Table 1). Peptide–DNA conjugates were synthesized on small (0.1–2 nmol) or preparative scales (greater than 10 nmol). For particularly difficult sequences or preparative-scale reactions, the coupling procedure was repeated multiple times to achieve high yields. In all cases, absolute yields for peptide–DNA products with *n* amino acids exceeded (0.9)*^n^*. Fmoc deprotection was carried out as described for succinimidyl ester coupling. See Supporting Information for a more detailed description of peptide coupling.

#### Side chain deprotection.

For Lys(Ns), Arg(Ns), Cys(S*t*Bu), and His(CNP), the column was washed with 3 ml of DMF and subsequently incubated for 30 min with 700 μl of DMF containing 500 mM BME and 250 mM DBU while submerged in a 60 °C H_2_O bath. The column was then washed with 3 ml of DMF and 12 ml of DEAE bind buffer. Lys(Ns) can also be deprotected quantitatively with a DMF solution containing 5% thiophenol and saturated K_2_CO_3_ at 37 °C for 90 min. These conditions deprotect Arg(Ns) inefficiently, and have not been tested for Cys(S*t*Bu) or His(CNP).

For Asp(*t*Bu), Glu(*t*Bu), and His(Trt), after HPLC purification, the *tert*-butyl ester and/or trityl containing oligonucleotide–peptide hybrid was incubated in a 20-mM MgCl_2_ solution at 70 °C, yielding quantitative deprotection in 3 h (Asp, His) or 12 h (Glu). Deprotection can alternatively be carried out before HPLC purification: after eluting from the DEAE Sepharose column, NaOAc (pH 5.2) and MgCl_2_ were added to final concentrations of 30 mM and 200 mM, respectively, and the solution was then incubated at 70 °C for the appropriate time. In contrast, acid deprotection on solid support was inefficient.

## Supporting Information

Figure S1 MALDI-MS Analysis of Conjugates All reported conjugates were verified by MALDI-MS analysis. Example mass spectra of a conjugate before (A; Leu-NC20) and after (B; Arg-Leu-NC20) peptide coupling. Calculated masses are noted to the left of the mass peaks.
(149 KB PDF).

Figure S2 Sequential Coupling Efficiencies and Yields of Peptide–DNA Synthesis (A) Reaction scheme for synthesis of GLFYG-NC20. Coupling efficiencies for individual steps are noted in black, and absolute yields from NC20 are noted in red. MALDI-MS results for all species are denoted under each species as “Observed (Calculated).” See Protocol S1 for precise coupling procedures.
(B) HPLC analysis of sequential couplings during peptide synthesis monitored at 260 nm. Load and elutes from columns 1–5 are indicated. Sequential coupling efficiencies (black) were calculated by integration of recovered aminated DNA peaks. Absolute yields (red) were calculated by integration of intended product peak relative to load. A nonaminated 10-base oligonucleotide (10mer) was included as a control for nonspecific DNA loss and modification. Percent recovery of 10mer is noted in red. The HPLC analysis employed a 60-min gradient of 0%–45% MeCN in100 mM TEAA (pH 5.5).
(368 KB PDF).

Protocol S1 Synthetic Methods (1.0 MB PDF).

## Abbreviations

BME: β-mercaptoethanol
CNP: 4-cyano-2-nitrophenyl
DBU: 1,8-diazabicyclo[5.4.0]undec-7-ene
DEAE: diethylaminoethyl
DMF: *N*,*N*-dimethylformamide
EDC: 1-(3-[dimethylamino]propyl)-3-ethylcarbodiimide hydrochloride
Fmoc: 9-fluorenylmethyoxycarbonyl
HOAt: 1-hydroxy-7-azabenzotriazole
HPLC: high performance liquid chromatography
MALDI-MS: matrix-assisted laser desorption/ionization mass spectrometry
MeOH: methanol
Ns: nosyl
PEG: polyethylene glycol
ssDNA: single-stranded DNA
*t*Bu: *tert*-butyl
Trt: trityl
