# Supplementary figures and images for: DNA Display III. Solid-Phase Organic Synthesis on Unprotected DNA
Source: PLoS Biol. 2004 Jun 22;2(7):e175. doi: 10.1371/journal.pbio.0020175 (PMC434150; doi:10.1371/journal.pbio.0020175)

A

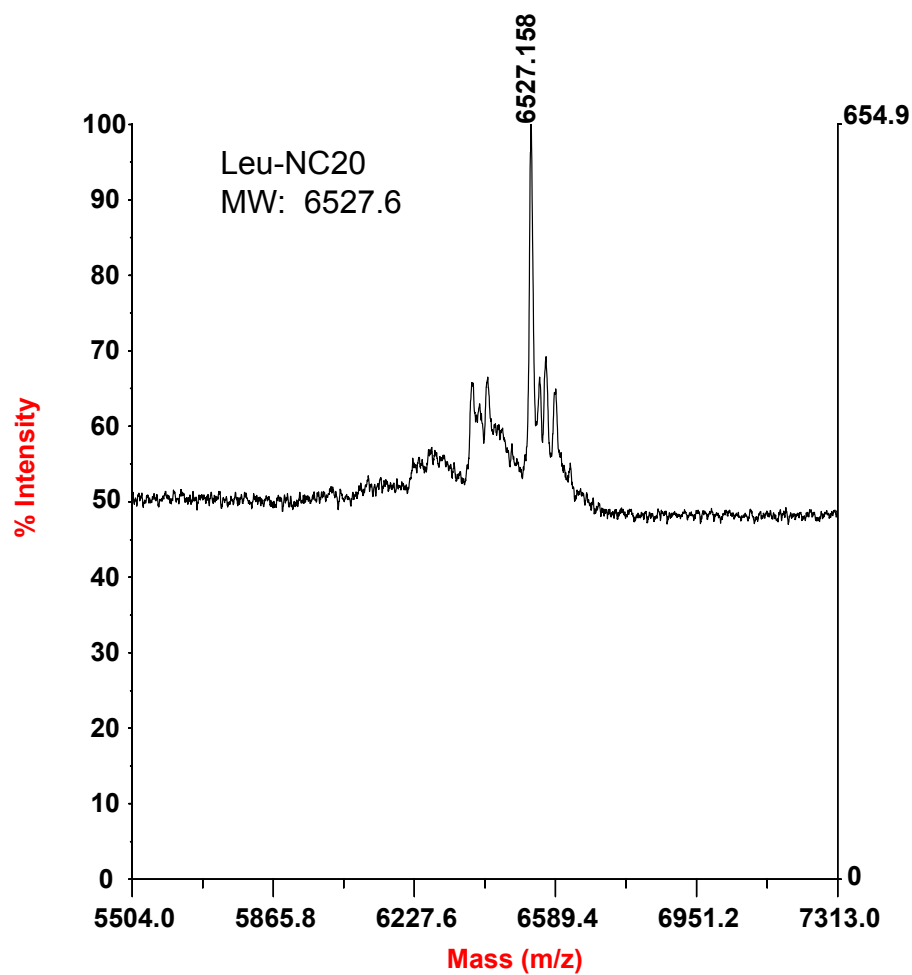

B

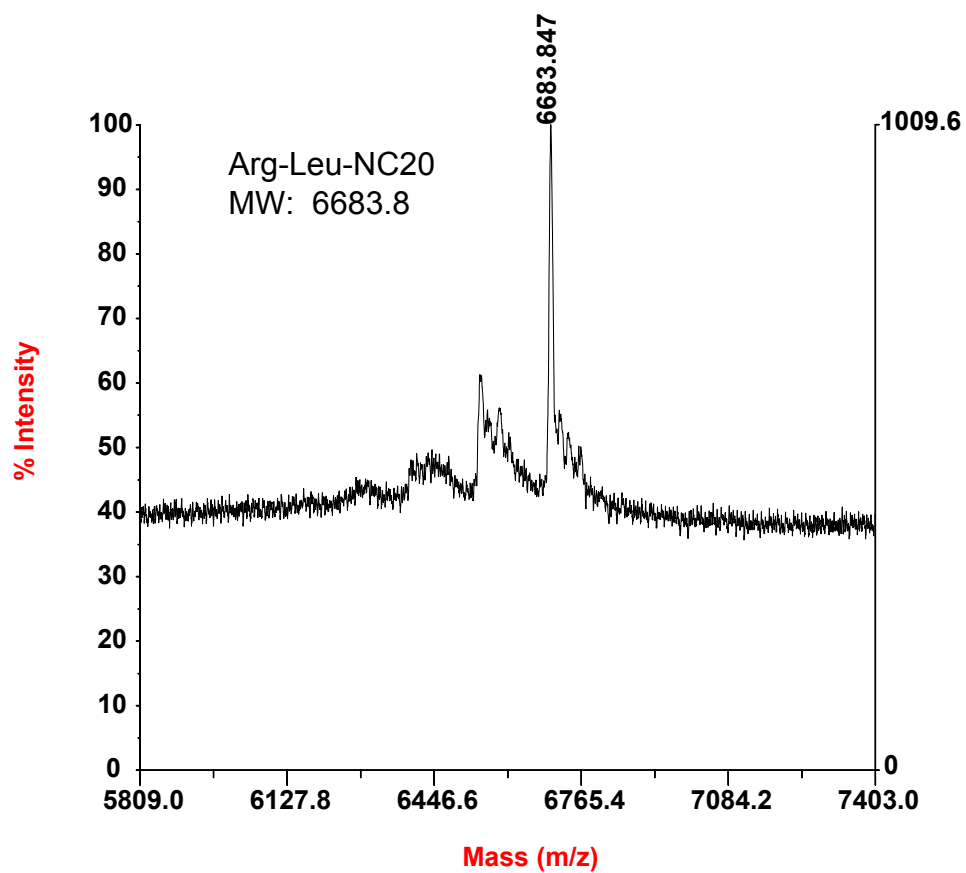

Supplement: Figure S1 — All reported conjugates were verified by MALDI-MS analysis. Example mass spectra of a conjugate before (A; Leu-NC20) and after (B; Arg-Leu-NC20) peptide coupling. Calculated masses are noted to the left of the mass peaks. (149 KB PDF). [file pbio.0020175.sg001.pdf]

A

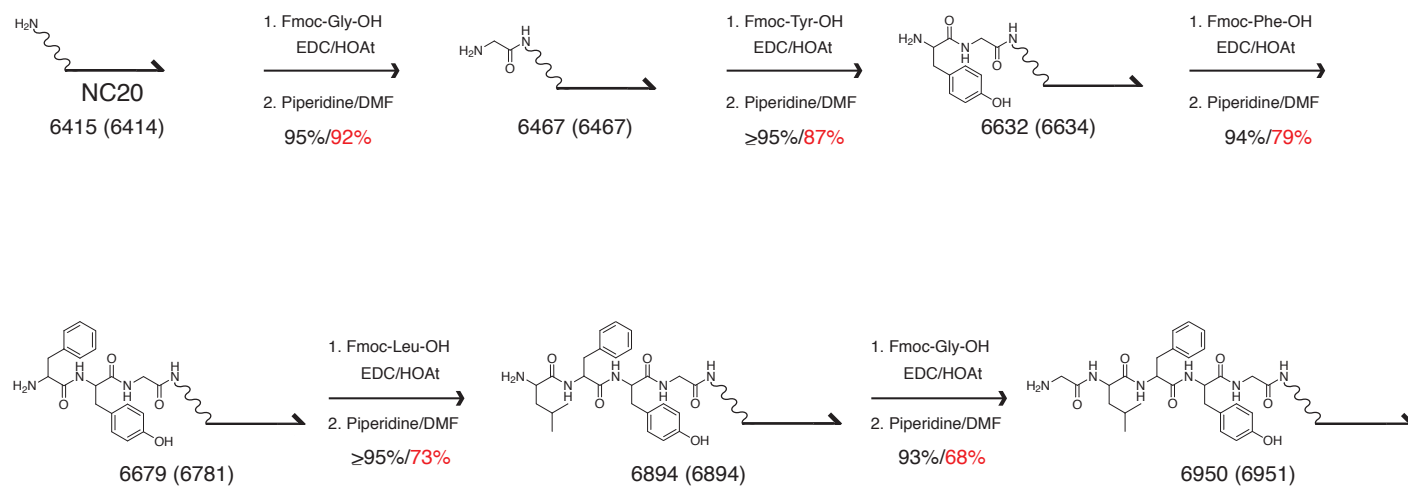

B

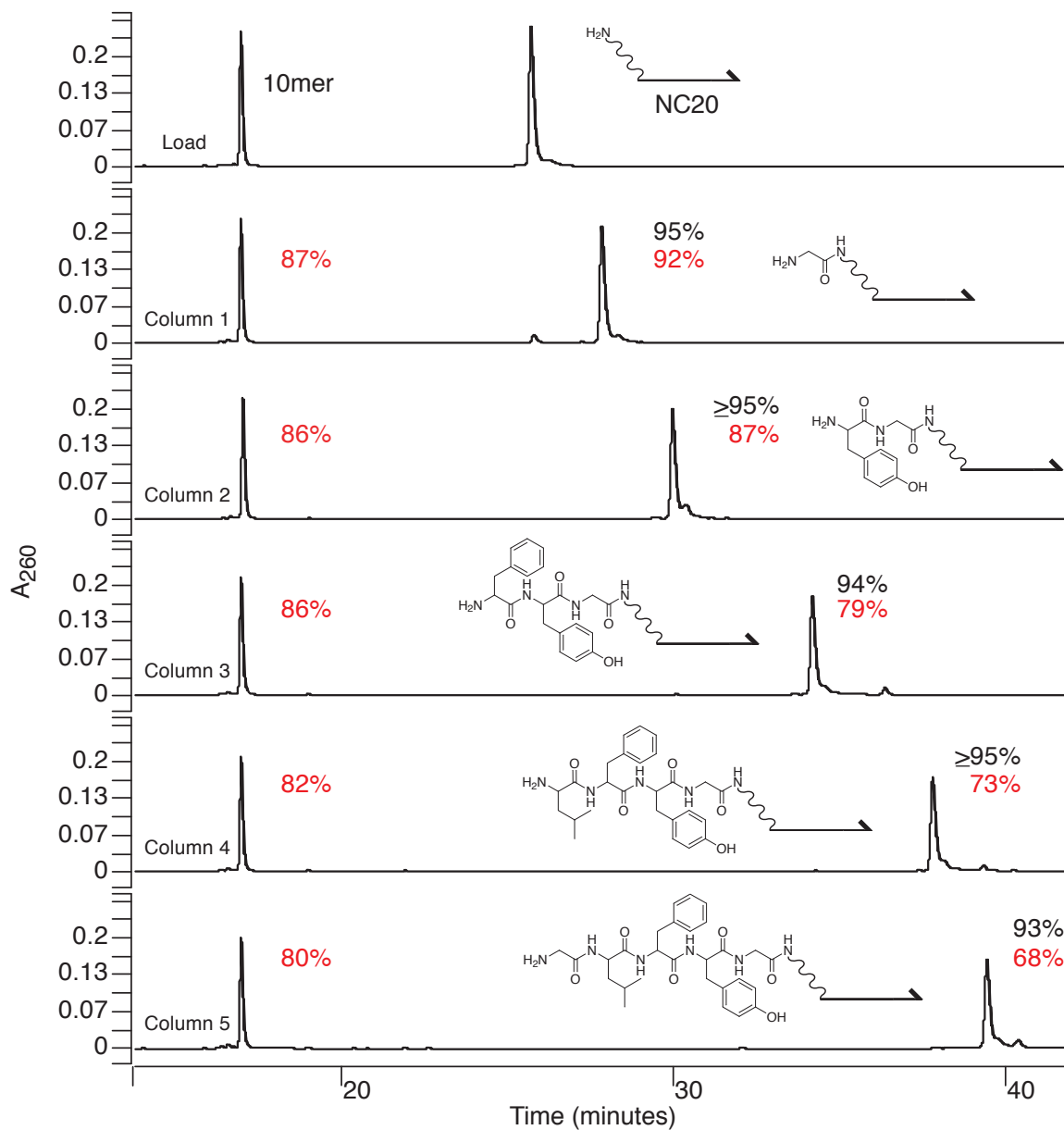

Supplement: Figure S2 — (A) Reaction scheme for synthesis of GLFYG-NC20. Coupling efficiencies for individual steps are noted in black, and absolute yields from NC20 are noted in red. MALDI-MS results for all species are denoted under each species as “Observed (Calculated).” See Protocol S1 for precise coupling procedures. (B) HPLC analysis of sequential couplings during peptide synthesis monitored at 260 nm. Load and elutes from columns 1–5 are indicated. Sequential coupling efficiencies (black) were calculated by integration of recovered aminated DNA peaks. Absolute yields (red) were calculated by integration of intended product peak relative to load. A nonaminated 10-base oligonucleotide (10mer) was included as a control for nonspecific DNA loss and modification. Percent recovery of 10mer is noted in red. The HPLC analysis employed a 60-min gradient of 0%–45% MeCN in100 mM TEAA (pH 5.5). (368 KB PDF). [file pbio.0020175.sg002.pdf]
