# Supplementary material for: DNA Display III. Solid-Phase Organic Synthesis on Unprotected DNA
Source: PLoS Biol. 2004 Jun 22;2(7):e175. doi: 10.1371/journal.pbio.0020175 (PMC434150; doi:10.1371/journal.pbio.0020175)
Supplement: Protocol S1 — (1.0 MB PDF). [file pbio.0020175.sd001.pdf]

Supporting Information

Peptide Synthesis Detailed protocol - GLFYG-NC20. Two nanomoles NC20 and 2 nanomoles of a non-aminated 10-base oligonucleotide were loaded onto each of 5 DEAE columns in 1 ml DEAE bind buffer using a vacuum manifold (Figure S2). Each column was washed with 3 ml of DEAE bind buffer, followed by 3 ml of water and 3 ml of MeOH. The columns were then removed from the manifold and placed between a 1 ml syringe (bottom) and a 1 ml syringe barrel (top).

Coupling reactions were carried out precisely as follows. A 50 mM stock solution of HOAt in MeOH was prepared (6.8 mg HOAt in 1 ml MeOH) and mixed well. For each coupling reaction, 25 μmoles of the appropriate Fmoc-amino acid and 25 μmoles of EDC•HCl were weighed out into separate microcentrifuge tubes. Amino acids were dissolved in 450 μl of the appropriate coupling solvent (MeOH for Gly, Leu, and Tyr; 4:5 DMF:MeOH for Phe), and 50 μl of the 50 mM HOAt is added. For each coupling, the 500 μl amino acid/HOAt solution was pipetted into a tube containing 25 μmoles EDC, mixed, and quickly pipetted into the open syringe barrel on top of the prepared DEAE column. The solution was pulled through the column with the lower syringe and the upper syringe barrel was quickly removed to insert its plunger. Using the lower syringe the solution was pushed back up through the column until all air was removed. The intact syringe was then added back to the top of the column, and the solution was pushed back and forth through the column several times. The reaction was then allowed to incubate at RT for 30 minutes, after which the column was moved back to the vacuum manifold and washed with 3 ml MeOH followed by 3 ml of DMF using a 3 ml syringe

barrel. 3 ml of 4:1 DMF:piperidine is added to the syringe barrel. 1.5 ml is pulled through the column, followed by a 3 minute incubation. An additional 1 ml is pulled through the column, followed by a 17 minute incubation. The remaining DMF:piperidine was pulled through the column. The column was then washed with 3 ml of DMF followed by 3 ml of MeOH before a subsequent coupling reaction.

Using these conditions the following conjugates were synthesized in parallel: G-NC20 (column 1), YG-NC20 (column 2), FYG-NC20 (column 3), LFYG-NC20 (column 4), and GLFYG-NC20 (column 5).

After all amino acid couplings on a column, it was washed with 3 ml of DEAE bind buffer using the vacuum manifold. It was then removed from the manifold, and the DNA was eluted into a 2 ml microcentrifuge tube with 2 ml DEAE elute buffer using a new 3 ml syringe. Elutes were analyzed by HPLC (Figure S2).

Succinimidyl-ester synthesis. Succinimidyl esters of amino acids were synthesized following standard methods (Anderson et al. 1963). Typically, 5 g Fmoc-amino acid and a three-fold molar excess of NHS were dissolved in 125 ml of DCM and 5 ml of DMF. A three fold molar excess of EDC was added in 125 ml DCM. The reaction mixture was stirred for 30 minutes at room temperature. After removing solvent under reduced pressure, 250 ml diethylether was added followed by 300 ml 0.1 M HCl. The reaction product was extracted into the organic layer, which was subsequently washed three times with 300 ml 0.1 M HCl, twice with saturated $NaHCO_3$, and three times with brine. The organic layer was then dried over Na_2SO_4, and solvent was removed by rotary evaporation, leaving a white solid. More polar amino acid products were extracted into EtOAc/diethylether mixtures. Activated amino acids were used with no further purification.

Fmoc-Asn-OSu synthesis was an exception. Fmoc-Asn-OH (3 g, 8.47 mmol), *N*-hydroxysuccimide (1.38 g, 12 mmol), and DCC (2.09 g,10.2 mmol) were added to 250 ml of freshly distilled dioxane. Reactants were not completely soluble at the onset of the reaction. The mixture was stirred at room temperature for 16 hours, at which time a white precipitate was present. After filtering, the solution was acidified with acetic acid and dry loaded onto silica. Purification over silica was carried out with a linear gradient from 10:89:1 dioxane/DCM/AcOH to 99:1 dioxane/AcOH over 8 column volumes. Fractions containing Fmoc-Asn-OSu were pooled, concentrated, redissolved in THF, and dried, yielding 3.0 grams of white solid (78% yield). ESI-MS: $[M+Na]^+$ 474.1 (474.1 calcd.).

Fmoc-Lys(Coumarin)-OH (1) synthesis.

2-[(4-methyl-2-oxo-*2H*-chromen-7-yl)oxy]acetyl chloride was synthesized as previously described (Chimichi et al. 2002). Fmoc-Lys-OH (4.23 g, 11.5 mmol) and DIEA (10 ml, 57.4 mmol) were dissolved in 240 ml of THF/H_2O (50/50) and added to a vessel containing 10 mmol of the coumarin acid chloride under nitrogen. The reaction was stirred at room temperature for one hour and transferred to a separation funnel. One volume of EtOAc was added, and the mixture was washed three times with 1 M citric acid and once with brine. The organic layer was dried over Na_2SO_4, and concentrated by rotary evaporation. The concentrate was loaded onto silica and purified with a linear gradient of 97:1:2 DCM/MeOH/AcOH to 95:3:2 DCM/MeOH/AcOH over 8.5 column volumes. Rotary evaporation of the product containing fractions yielded 2.00 grams of a yellow solid (34% yield). ESI-MS: $[M+H]^+$ 585.1 (585.2 calcd), $[M-H]^-$ 583.0 (583.2 calcd). ^{1}H NMR(400MHz, Acetone-d_6): δ 7.84 (d, *J* = 1.2 Hz, 2H), 7.72-7.64 (m, 3H), 7.42-7.29 (m, 4H), 6.96 (dd, *J* = 8.0, 2.8 Hz, 1H), 6.88 (d, J = 2.4 Hz, 1H), 6. 15 (m, 1H),

4.59 (s, 1H), 4.33-4.30 (m,2H), 4.23-4.18(m, 2H), 3.32 (t, J = 6.8 Hz, 2H), 2.41 (m, 3H), 1.95-1.72 (m, 2H), 1.63-1.43 (m, 4H). ^{13}C NMR (100MHz, DMSO-d_6): δ 174.1, 166.8, 160.7, 160.1, 156.2, 154.5, 153.4, 143.9, 142.7, 127.7, 127.1, 126.4, 125.3, 120.1, 113.0, 112.4, 111.5, 101.8, 67.2, 65.6, 53.9, 46.7, 38.2, 30.5, 28.7, 23.1, 18.2. See Figure S3A for UV absorption spectrum.

Fmoc-Lys(Ns)-OH (2) synthesis. Fmoc-Lys-OH (0.20 g, 0.543 mmol) was dissolved in 20 ml of 50/50 THF/H_2O at room temperature. DIEA (870 μl, 5 mmol) was added to the stirring solution, followed by 2-nitrobenzenesulfonyl chloride (0.135 g, 0.6 mmol). After 30 minutes, the reaction mixture was transferred to a separation funnel containing 20 ml of EtOAc and 20 ml of 1 M citric acid. The organic layer was washed four times with 1 M citric acid and once with brine, dried over Na_2SO_4, and rotary evaporated, leaving 0.29 grams of a white-yellow solid (96% yield). ESI-MS: $[M\text{-}H]^-$ 552.0 (552.2 calcd.). ^{1}H NMR(400MHz, DMSO-d_6): δ 8.08 (t, J = 5.4 Hz, 1H), 7.99-7.93 (m, 2H), 7.90-7.81 (m, 4H), 7.72-7.70 (m, 2H), 7.60-7.57 (m, 1H), 7.42-7.29 (m, 4H), 4.28-4.26 (m, 2H), 4.23-4.19 (m, 1H), 3.90-3.82 (m, 1H), 2.87 (dd, J = 12.8, 6,8 Hz, 2H), 1.66-1.46 (m, 2H), 1.45- 1.20 (m, 4H). ^{13}C NMR (75MHz, DMSO-d_6): δ 174.0, 156.2, 147.8, 143.8, 140.7, 134.0, 132.8, 132.6, 129.4, 127.7, 127.1, 125.3, 124.4, 120.1, 65.6, 53.7, 46.7, 42.5, 30.2, 28.8, 22.7. See Figure S3B for UV absorption spectrum.

Fmoc-Arg(Ns)-OH (3) synthesis. Boc-Arg-OH (5.0 g, 18.23 mmol) was dissolved in 80 ml of acetone and 20 ml of H_2O at 0°C. 5.5 ml of 10 M NaOH was added to basify the solution. 2-nitrobenzenesulfonyl chloride (8.5 g, 37.77 mmol) was added, and the solution was stirred at 0°C for 2 hours with occasional drop-wise addition of 4 M NaOH to maintain a pH of 11 to 11.5. The reaction mixture was then neutralized with a 10% citric acid solution, and the acetone was removed by rotary evaporation. The

remaining aqueous layer was washed three times with 60 ml of diethylether, acidified with 10% citric acid to pH 3, and extracted three times with 80 ml of EtOAc. The combined extracts were washed once with 100 ml of 10% citric acid and twice with 100 ml of brine. After drying the organic layer over Na_2SO_4, rotary evaporation yielded a yellow solid (6.1 g, 73% yield). To remove the *t*-butyl carbamate group, the solid was dissolved in 20 ml of trifluoroacetic acid and stirred at room temperature for 2 hours. Subsequent rotary evaporation gave a dark yellow solid (6.3 g). A portion of the presumed Arg(Ns)-OH•TFA salt (1.35 g, 2.85 mmol) was dissolved in 10.77 ml of aqueous 0.85 M Na_2CO_3 in an ice bath. Fmoc-succinimide (1.47, 4.35 mmol) was added to the stirring solution in 10 ml of DMF. Five minutes after the addition, the ice bath was removed and an additional 5 ml of DMF was added. The mixture was stirred for an additional two hours at room temperature, then acidified to pH 2 with 1 M HCl, yielding a dark yellow precipitate. After decanting, the solid was dissolved in 100 ml of EtOAc and washed with 0.1 M HCl and brine. Drying over Na_2SO_4 and rotary evaporation yielded a pale yellow solid (1.60 g, 70% yield from Boc-Arg-OH). ESI-MS: $[M+Na]^+$ 604.1 (604.2 calcd.), $[M-H]^-$ 580.0 (580.2 calcd.). ^{1}H NMR(400MHz, DMSO-d_6): δ 7.98-7.85 (m, 4H), 7.78-7.66 (m, 4H), 7.41-7.28 (m, 4H), 4.21-4.18 (m, 3H), 3.74 (m, 1H), 1.72-1.39 (m, 4H). ^{13}C NMR (75MHz, DMSO-d_6): δ 175.1, 162.4, 155.7, 147.4, 144.0, 140.7, 132.6, 131.7, 128.8, 127.6, 127.1, 125.3, 123.6, 121.4, 120.1, 65.5, 55.0, 46.7, 35.8, 30.8, 25.7. See Figure S3C for UV absorption spectrum.

Fmoc-His(CNP)-OH (4) Synthesis. Fmoc-His-OH (0.926 g, 2.45 mmol) and 4-fluoro-3-nitro-benzonitrile (0.448 g, 2.70 mmol) were dissolved in 10 ml of DMSO and stirred at room temperature for 3 hours. H_2O (50 ml) and 1 M citric acid (50 ml) were added to the solution, producing a yellow precipitate. The mixture was extracted three

times with 100 ml of EtOAc. The combined organic layers were washed once with 300 ml of 1 M citric acid and twice with 300 ml of brine, dried over Na_2SO_4, and concentrated to approximately 10 ml. Upon addition of the concentrated solution to diethyl ether, a yellow precipitate formed and was isolated by filtration. Drying yielded 0.90 g of a yellow solid (70% yield). ESI-MS: $[M+H]^+$ 524.1 (524.1 calcd). ^{1}H NMR(400MHz, Acetone-d_6): δ 8.65 (d, *J* = 2.0 Hz, 1H), 8.29 (dd, *J* = 8, 1.6 Hz, 1H), 8.16 (s, 1H), 7.95 (d, *J* = 8.4Hz, 1H), 7.84 (d, *J* = 7.6 Hz, 2H), 7.70 (m, 2H), 7.41-7.27 (m, 5H), 4.66-4.61 (m, 1H), 4.34-4.19 (m, 3H), 3.29-3.14 (m, 2H). ^{13}C NMR (75MHz, Acetone-d_6): δ 173.0, 162.7, 156.8, 145.4, 145.0, 142.0, 130.0, 138.4, 137.7, 134.5, 130.5, 128.5, 127.9, 126.2, 120.7, 118.6, 116.8, 113.9, 67.2, 55.0, 47.9, 31.0. See Figure S3D for UV absorption spectrum.

Additional Reference

Chimichi S, Boccalini M, Cosimelli B (2002) A new convenient route to 2-oxoethoxycoumarins: Key intermediates in the synthesis of natural products. Tetrahedron 58: 4851–4858.

Figure S3. UV spectra of novel amino acids

A) Fmoc-Lys(Coumarin)-OH. 10 μg/ml in acetonitrile.

B) Fmoc-Lys(Ns)-OH. 10 μg/ml in acetonitrile.

C) Fmoc-Arg(Ns)-OH. 10 μg/ml in methanol.

D) Fmoc-His(CNP)-OH. 10 μg/ml in methanol.

A

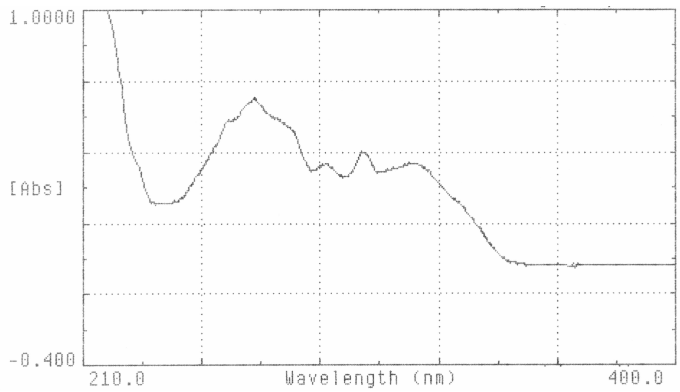
1.0000
[Abs]
-0.400
210.0
Wavelength (nm)
400.0

B

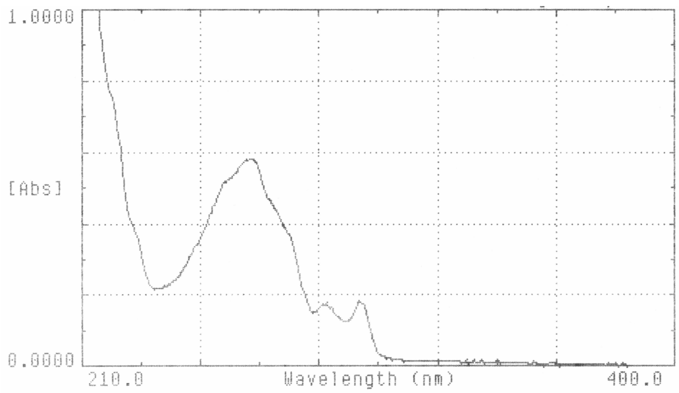
1.0000
[Abs]
0.0000
210.0
Wavelength (nm)
400.0

C

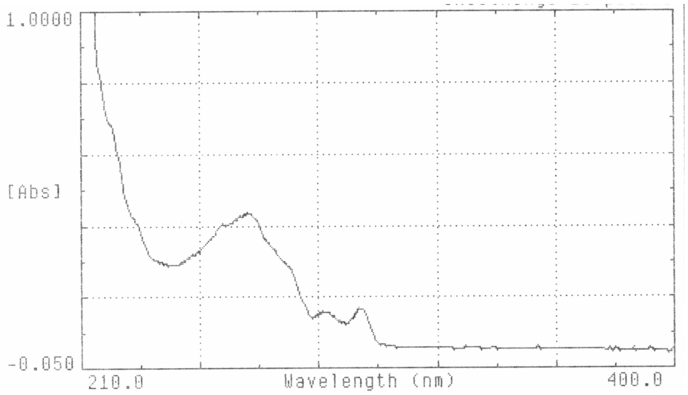
1.0000
[Abs]
-0.050
210.0
Wavelength (nm)
400.0

D

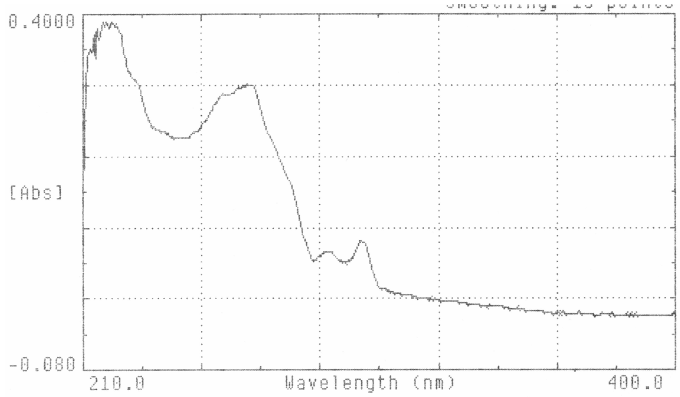
0.4000
[Abs]
-0.080
210.0
Wavelength (nm)
400.0
